# Wnt status-dependent oncogenic role of BCL9 and BCL9L in hepatocellular carcinoma

**DOI:** 10.1007/s12072-019-09977-w

**Published:** 2019-08-22

**Authors:** Nicole Huge, Maria Sandbothe, Anna K. Schröder, Amelie Stalke, Marlies Eilers, Vera Schäffer, Brigitte Schlegelberger, Thomas Illig, Beate Vajen, Britta Skawran

**Affiliations:** Department of Human Genetics, Hannover Medical School, Carl-Neuberg-Straße 1, Hannover, 30625 Germany

**Keywords:** Liver cancer, B9L, BCL9-2

## Abstract

**Background:**

Activation of Wnt/β-catenin pathway is a frequent event in hepatocellular carcinoma and is associated with enhanced cell survival and proliferation. Therefore, targeting this signaling pathway is discussed as an attractive therapeutic approach for HCC treatment. BCL9 and BCL9L, two homologous coactivators of the β-catenin transcription factor complex, have not yet been comprehensively characterized in HCC. We aimed to elucidate the roles of BCL9 and BCL9L, especially regarding Wnt/β-catenin signaling and their prognostic value in HCC.

**Methods:**

Expression of BCL9/BCL9L was determined in HCC cell lines (HLE, HLF, Huh7, HepG2, Hep3B, and Huh6) and normal liver cell lines (THLE-2 and THLE-3). To analyze proliferation and apoptosis, BCL9 and/or BCL9L were knocked down in Wnt-inactive HLE and Wnt-active HepG2 and Huh6 cells using siRNA. Subsequently, Wnt reporter assays were performed in HepG2 and Huh6 cells. BCL9 and BCL9L expression, clinicopathological and survival data of public HCC datasets were analyzed, taking the Wnt signaling status into account.

**Results:**

Knockdown of BCL9L, but not of BCL9, reduced Wnt signaling activity. Knockdown of BCL9 and/or BCL9L reduced cell viability and increased apoptosis of Wnt-inactive HCC cells, but had no effect in Wnt-active cells. Expression of *BCL9* and *BCL9L* was upregulated in human HCC and increased with progressing dedifferentiation. For *BCL9L*, higher expression was observed in tumors of larger size. Overexpression of *BCL9* and *BCL9L* correlated with poor overall survival, especially in HCC without activated Wnt signaling.

**Conclusion:**

Oncogenic BCL9 proteins represent promising targets for cancer therapy and inhibiting them may be particularly beneficial in Wnt-inactive HCCs.

**Graphic abstract:**

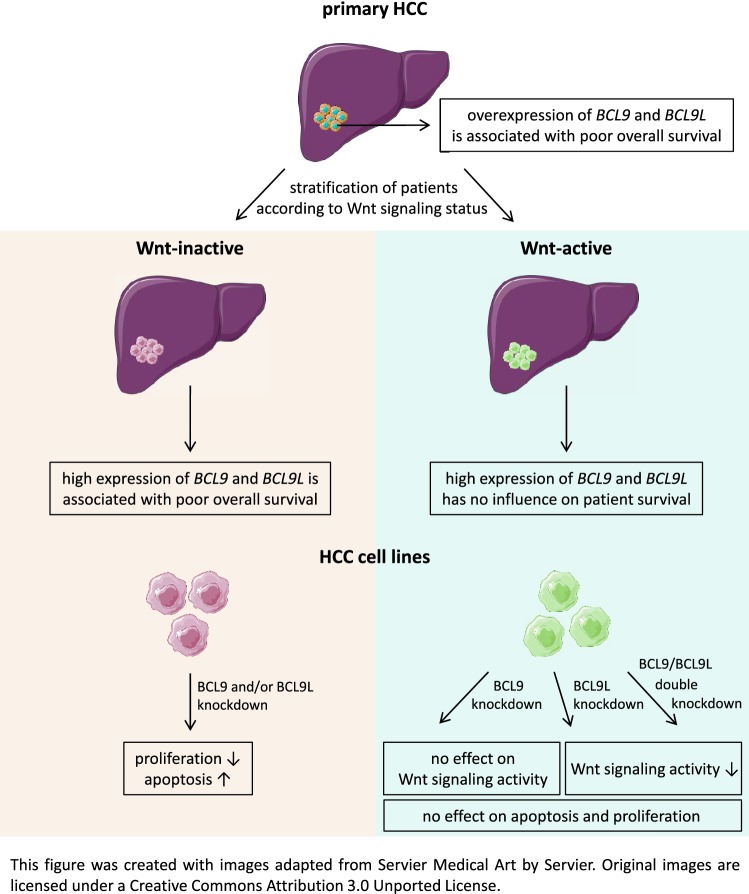

**Electronic supplementary material:**

The online version of this article (10.1007/s12072-019-09977-w) contains supplementary material, which is available to authorized users.

## Introduction

Hepatocellular carcinoma (HCC) accounts for about 80% of primary liver cancers and is the second most common cause of cancer-related death worldwide [1]. Risk factors for HCC development include chronic hepatitis B and C virus infections, exposure to aflatoxin B1, alcohol abuse, and the presence of metabolic syndrome. If diagnosed early, HCC can successfully be cured by surgical resection or liver transplantation. However, HCC is mostly diagnosed at an advanced stage when palliative approaches remain the only treatment option. To date, the multi-kinase inhibitors sorafenib and lenvatinib are the only targeted first line options for HCC therapy. Both have shown significant, although moderate, efficiency in patients with advanced HCC [2, 3]. Thus, the characterization of tumor-related signaling pathways, the identification of potential molecular targets and the development of targeted therapies are still major goals of HCC research.

Disrupted Wnt/β-catenin signaling is a common hallmark of HCC. This predominantly results from mutations in the *CTNNB1* gene coding for β-catenin, which are found in 37% of patients [4]. Deregulation of the Wnt/β-catenin pathway is an early event in HCC development and has been associated with an aggressive HCC phenotype [5]. Upon activation of canonical Wnt signaling, β-catenin accumulates in the cytoplasm followed by its translocation into the nucleus. There, it forms a transcriptionally active complex with TCF/LEF transcription factors, Pygo, and one of the coactivators BCL9 (B-cell CLL/lymphoma 9) or BCL9L (B-cell CLL/lymphoma 9 like) resulting in activation of downstream target genes.

Oncogenic function of the homologs BCL9 and BCL9L is well characterized in colon cancer, where BCL9 proteins are frequently overexpressed [6, 7]. There, BCL9 enhances proliferation and the invasive and metastatic potential by promoting β-catenin-dependent transcription [6]. In HCC, overexpression of BCL9 is associated with microvascular invasion, intrahepatic metastasis, and poor prognosis [8]. In normal liver, BCL9 expression has not been detected [8, 9]. BCL9L promotes tumor growth and local invasion in mouse intestinal tumors by increasing the expression of canonical Wnt target genes [7]. While constitutive deletion of *Bcl9* and *Bcl9l* causes embryonic lethality [10], a liver-specific combined *Bcl9/Bcl9l* deletion does not perturb normal liver homeostasis or proliferation in mice [11]. However, in hepatocellular tumors driven by mutant β-catenin, the deletion of *Bcl9/Bcl9l* has resulted in increased survival rates of mice, a reduced liver-to-body weight ratio, diminished cell proliferation, and a decreased expression of Wnt target genes [11].

In the context of HCC, the role of BCL9 and specifically of BCL9L has not been characterized in detail. Therefore, we aimed to elucidate the roles of BCL9 and BCL9L, especially regarding Wnt/β-catenin signaling, by performing BCL9 and/or BCL9L knockdown experiments and Wnt reporter assays in HCC cell lines. As we were interested in the prognostic value of *BCL9* and particularly *BCL9L* expression in HCC, we analyzed survival data of public HCC data sets taking into account the Wnt signaling status of patients.

## Materials and methods

### Cell culture and transfection

HCC cell lines (HLE, HLF, Huh7, HepG2, Hep3B, and Huh6) were cultured in Dulbecco’s Modified Eagle Medium with 10% fetal calf serum (FCS), 2 mM l-glutamine, and 100 U/mL penicillin/streptomycin. Normal liver cell lines (THLE-2 and THLE-3) were cultured in BEGM (Lonza, Basel, Switzerland) without gentamycin/amphotericin and epinephrine, but with additional 70 ng/mL phosphoethanolamine, 5 ng/mL epidermal growth factor, and 10% FCS. For siRNA-mediated knockdown, cells were transfected with Silencer Select siRNAs (Thermo Fisher Scientific, Waltham, MA, USA) using HiPerFect Transfection Reagent (Qiagen, Hilden, Germany). For single or double knockdown, cells were transfected with siRNAs against *BCL9* (s1936, s1937) and/or *BCL9L* (s49114, s49115). AllStars Negative Control siRNA (Qiagen) was used as non-targeting control siRNA. For activation of the Wnt signaling pathway, cells were treated with 20 mM lithium chloride (LiCl) [or sodium chloride (NaCl), as control] [12] or with 1 µM CHIR99021 [or dimethyl sulfoxide (DMSO), as vehicle control] [13] for 24 h.

### Quantitative real-time PCR

For quantification of mRNA expression levels, total RNA was isolated with Direct-zol RNA MiniPrep Kit (Zymo Research, Irvine, CA, USA). RNA was transcribed into cDNA using the High-Capacity cDNA Reverse Transcription Kit (Thermo Fisher Scientific). Relative mRNA expression was measured in triplicates using Taqman Gene Expression Assays (Thermo Fisher Scientific) against *BCL9* (Hs00979216_m1), *BCL9L* (Hs0069441_m1), *BAMBI* (Hs03044164_m1), and *AXIN2* (Hs00610344_m1). *TBP* (Hs00920494_m1) was used as reference gene for normalization.

### Western blot

For protein analysis, whole-cell lysates were prepared with RIPA buffer employing equal cell numbers per sample. Protein levels were analyzed using a standard western blot protocol with antibodies against BCL9 (AF3996; R&D Systems, Wiesbaden, Germany), BCL9L (ab113110; Abcam, Cambridge, UK), β-catenin (ABIN104390; antibodies-online, Aachen, Germany), and β-actin (3700; Cell Signaling, Leiden, The Netherlands).

### Cell viability and apoptosis assays

Cell viability and apoptosis were measured in triplicate every 24 h using WST-1 Proliferation Reagent (Roche, Basel, Switzerland) and the Caspase3/7 Glo Assay (Promega, Madison, WI, USA), respectively.

### Wnt reporter assay

Wnt/β-catenin signaling activity was assayed by pGL3-OT and pGL3-OF firefly luciferase reporter plasmids (gifts from B. Vogelstein) containing three copies of wild-type and mutant TCF4-binding sites, respectively [14]. Cells were transfected with pGL3-OT, pGL3-OF, or pGL3-basic control vector (Promega) using Lipofectamine 2000 (Thermo Fisher Scientific). For normalization, pGL4.70 (Promega) containing an EF1α promoter was co-transfected in all conditions. HepG2 and Huh6 cells were additionally transfected with siBCL9 and/or siBCL9L. Luciferase activities were measured 24 h after transfection in HLE and 48 h after transfection in HepG2 and Huh6 cells with the DualGlo Luciferase Assay System (Promega).

### Analysis of public data sets

For analysis of *BCL9* and *BCL9L* expression in non-tumorous liver and HCC tissue, we downloaded expression levels of NCBI GEO data sets GSE22058 and GSE25097 (https://www.ncbi.nlm.nih.gov/geo/). In addition, we downloaded log2-transformed, normalized mRNA expression values (RSEM, Illumina HiSeq_RNASeqV2) and clinicopathological data of the TCGA-LIHC cohort from the Cell Index Database CELLX [15] and the GDC portal (https://portal.gdc.cancer.gov/), respectively.

For survival analysis of the TCGA-LIHC cohort, we downloaded survival data together with *BCL9* and *BCL9L* expression levels from http://www.oncolnc.org/ [16]. *BCL9* and *BCL9L* expression was classified as high (upper median) or low (lower median) and, for combined survival analysis, patients were grouped into the following *BCL9*–*BCL9L* expression groups: high–high, high–low, low–high, and low–low. Furthermore, we stratified the TCGA-LIHC cohort into cases without or with Wnt/β-catenin signaling activation according to Sanchez-Vega et al. [17].

Microarray-derived robust multi-array average (RMA)-normalized expression data for *BCL9*, *BCL9L*, and *AXIN2* were downloaded from the Cancer Cell Line Encyclopedia (https://portals.broadinstitute.org/ccle) [18].

### Statistics

Data are represented as mean ± standard deviation of at least three independent experiments. Statistical significance was determined with the GraphPad Prism software (GraphPad Software, La Jolla, CA, USA) by two-tailed Student’s *t* tests or by one-way ANOVA with Dunnett’s multiple comparison test and Tukey’s multiple comparison test. Statistical significance of combined *BCL9*–*BCL9L* survival analysis was determined by log-rank test with Benjamini–Hochberg correction for multiple testing.

## Results

### *BCL9* and *BCL9L* are overexpressed in human HCC

**Fig. 1:**
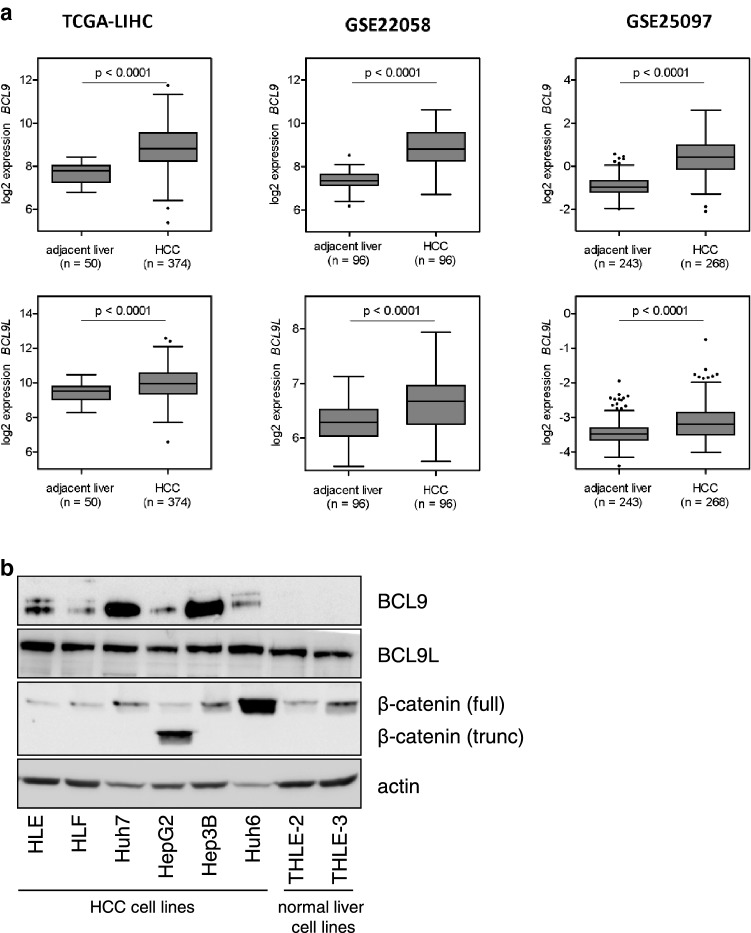
*BCL9* and *BCL9L* are overexpressed in human HCC. **a***BCL9* and *BCL9L* expression levels were analyzed using the three public HCC data sets TCGA-LIHC, GSE22058, and GSE25097. *BCL9* and *BCL9L* expression was significantly higher in HCC tissue than in adjacent non-tumorous liver tissue. Tukey box-and-whisker plot; two-tailed Student’s *t* test. **b** Western blot analysis of HCC cell lines (HLE, HLF, Huh7, HepG2, Hep3B, and Huh6) and immortalized liver cell lines (THLE-2, THLE-3) for BCL9, BCL9L, and β-catenin protein expression. HepG2 and Huh6 harbor an activating β-catenin deletion and mutation, respectively, causing an accumulation of β-catenin

In the context of HCC, only few studies on BCL9 and BCL9L have been published. Therefore, we decided to characterize their effects in HCC in detail. We first analyzed the impact of *BCL9* and *BCL9L* in human HCC tissue to elucidate them in vivo relevance. We analyzed *BCL9* and *BCL9L* expression levels in three public HCC data sets: TCGA-LIHC, GSE22058, and GSE25097 (Fig. 1a). In all three data sets, *BCL9* and *BCL9L* expression levels were significantly higher in HCC tissue than in adjacent non-tumorous liver tissue. Our results show that both *BCL9* and *BCL9L* are frequently overexpressed in human HCC.

To establish suitable cell lines for the analysis of BCL9 and BCL9L expression, we tested six HCC cell lines (HLE, HLF, Huh7, HepG2, Hep3B, and Huh6) and two normal liver cell lines (THLE-2 and THLE-3) for BCL9, BCL9L, and β-catenin protein expression (Fig. 1b). For BCL9, all tested HCC cell lines showed moderate or strong expression, while no expression was detected in the two normal liver cell lines. For BCL9L, expression was detected in all HCC and normal liver cell lines. Moreover, we detected high β-catenin expression in HepG2 and Huh6 cells, as they harbor an activating β-catenin deletion and mutation, respectively. According to the Cancer Cell Line Encyclopedia (https://portals.broadinstitute.org/ccle), none of the cell lines harbors a mutation in *BCL9* or *BCL9L*. These cell lines are, therefore, suitable for further functional characterization of BCL9 and BCL9L.

### *BCL9* and *BCL9L* overexpression in human HCC correlates with poor overall survival

**Fig. 2:**
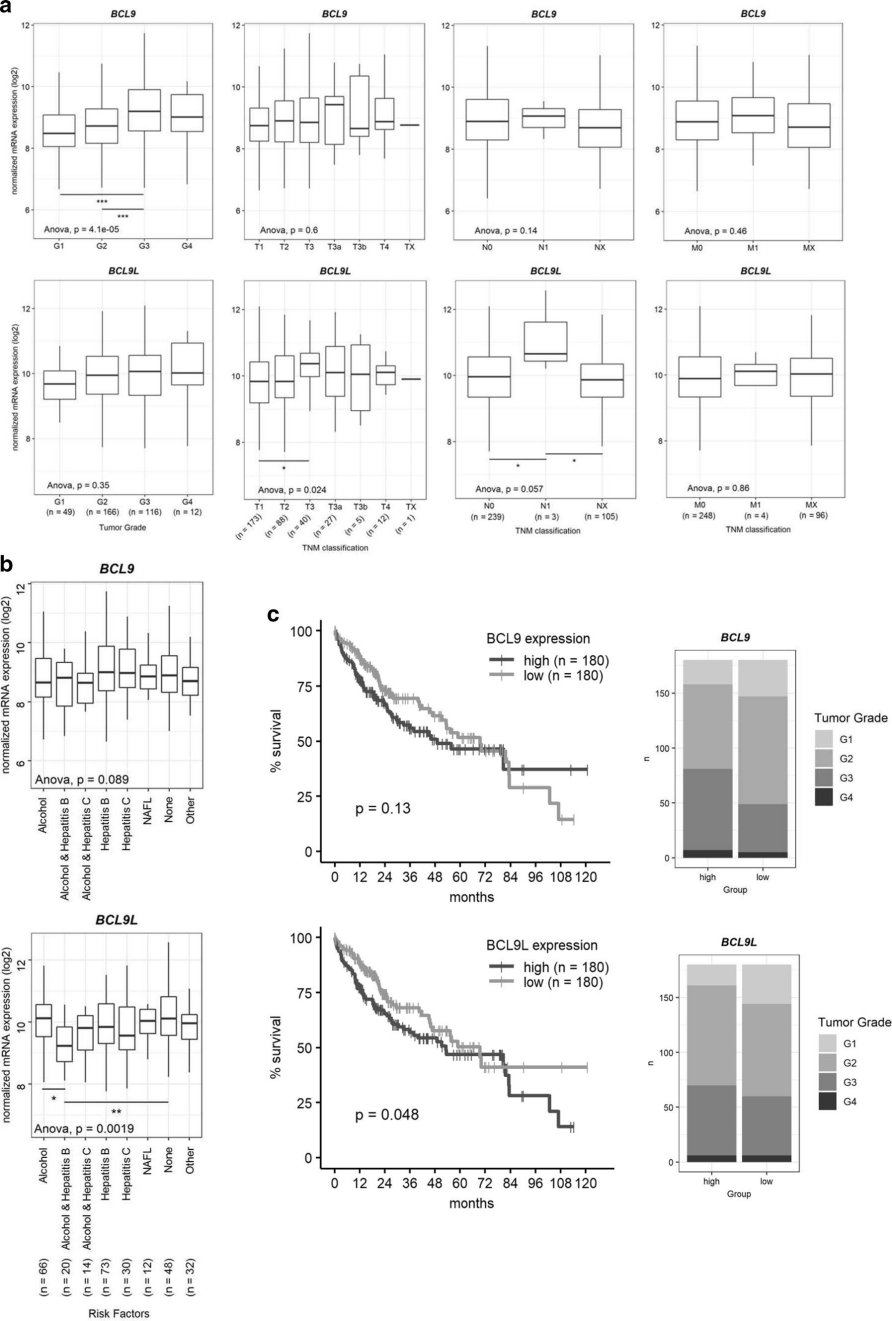
*BCL9* and *BCL9L* overexpression correlates with poor survival of HCC patients. **a***BCL9* and *BCL9L* expression levels of the TCGA-LIHC cohort were divided according to histologic tumor grade and TNM classification parameters [27]. Data are represented as Tukey’s box-and-whisker plot. *p < 0.05, ***p < 0.001; 1-way ANOVA with Tukey’s multiple comparison test. **b***BCL9* and *BCL9L* expression levels of the TCGA-LIHC cohort were divided according to tumor etiology. Data are represented as Tukey’s box-and-whisker plot. *p < 0.05, **p < 0.01; one-way ANOVA with Tukey’s multiple comparison test. **c***BCL9* and *BCL9L* expression values and survival data of the TCGA-LIHC cohort were retrieved from http://www.oncolnc.org/ [16]. Patients were grouped into low (lower median) or high (upper median) expressions of *BCL9* or *BCL9L*. Kaplan–Meier with log-rank test

To gain further insight into the in vivo relevance of *BCL9* and *BCL9L*, we analyzed their expression regarding clinicopathological parameters of the TCGA-LIHC cohort. Thereby, we identified an increased *BCL9* and *BCL9L* expression in high-grade compared to low-grade HCCs (Fig. 2a). For *BCL9L*, higher expression was observed in tumors of larger size and in HCCs containing cancer cells in nearby lymph nodes (Fig. 2a). While expression of *BCL9* seemed to be independent of tumor etiology, *BCL9L* levels were lowest in patients with alcohol abuse and hepatitis B infections (Fig. 2b). We also analyzed the impact of *BCL9* and *BCL9L* expression on survival of HCC patients in the TCGA-LIHC cohort (Fig. 2c). HCC patients with high *BCL9* or *BCL9L* expression showed a tendency for a worse overall survival in comparison to those with low expression. High expression levels of both *BCL9* and *BCL9L* also correlated with poor overall survival of HCC patients (Supp. Figure 1). Our results suggest that *BCL9* and *BCL9L* overexpression may contribute to a poor prognosis.

### Expression of *BCL9* and *BCL9L* is independent of Wnt signaling activation in liver cancer cells

In colon carcinoma, Wnt responsiveness of *BCL9* and *BCL9L* themselves has been proposed [12]. Based on our analysis of CCLE data, expression of *BCL9* and *BCL9L* is not increased in Wnt-active HepG2 and Huh6 cells compared to Wnt-inactive HCC cell lines (Supp. Figure 2a). Activation of Wnt signaling by LiCl or CHIR99021 treatment did not enhance the expression of *BCL9* and *BCL9L* in HCC cell lines (Supp. Figure 2b). Moreover, there is no evidence for an increase in *BCL9* or *BCL9L* expression in Wnt-active primary HCCs compared to Wnt-inactive HCCs in TCGA-LIHC data (Supp. Figure 2c). As positive control, *AXIN2*, a well-known Wnt-responsive gene, was clearly overexpressed in Wnt-active HepG2 and Huh6 cells as well as in Wnt-active primary HCCs (Supp. Figure 2a, c). In Wnt-inactive HLE cells, *AXIN2* expression was induced after activation of the Wnt signaling pathway (Supp. Figure 2b). Our results confirm Wnt responsiveness of *AXIN2*, while *BCL9* and *BCL9L* seem not to be Wnt-responsive in liver cancer cells.

### *BCL9L* knockdown decreases Wnt/β-catenin signaling in HCC cell lines

To further characterize the effects of BCL9 and BCL9L in HCC, we established siRNA-mediated knockdown of either BCL9 or BCL9L alone and a BCL9/BCL9L double knockdown in HLE, HepG2, and Huh6 cells (Supp. Figure 3). Successful knockdown of BCL9 and BCL9L was verified with qRT–PCR (Supp. Figure 3a) and western blotting (Supp. Figure 3b). Interestingly, knockdown of BCL9 led to an increase of BCL9L mRNA and protein levels and, to a lesser extent, vice versa. Protein expression of β-catenin was not affected.

**Fig. 3:**
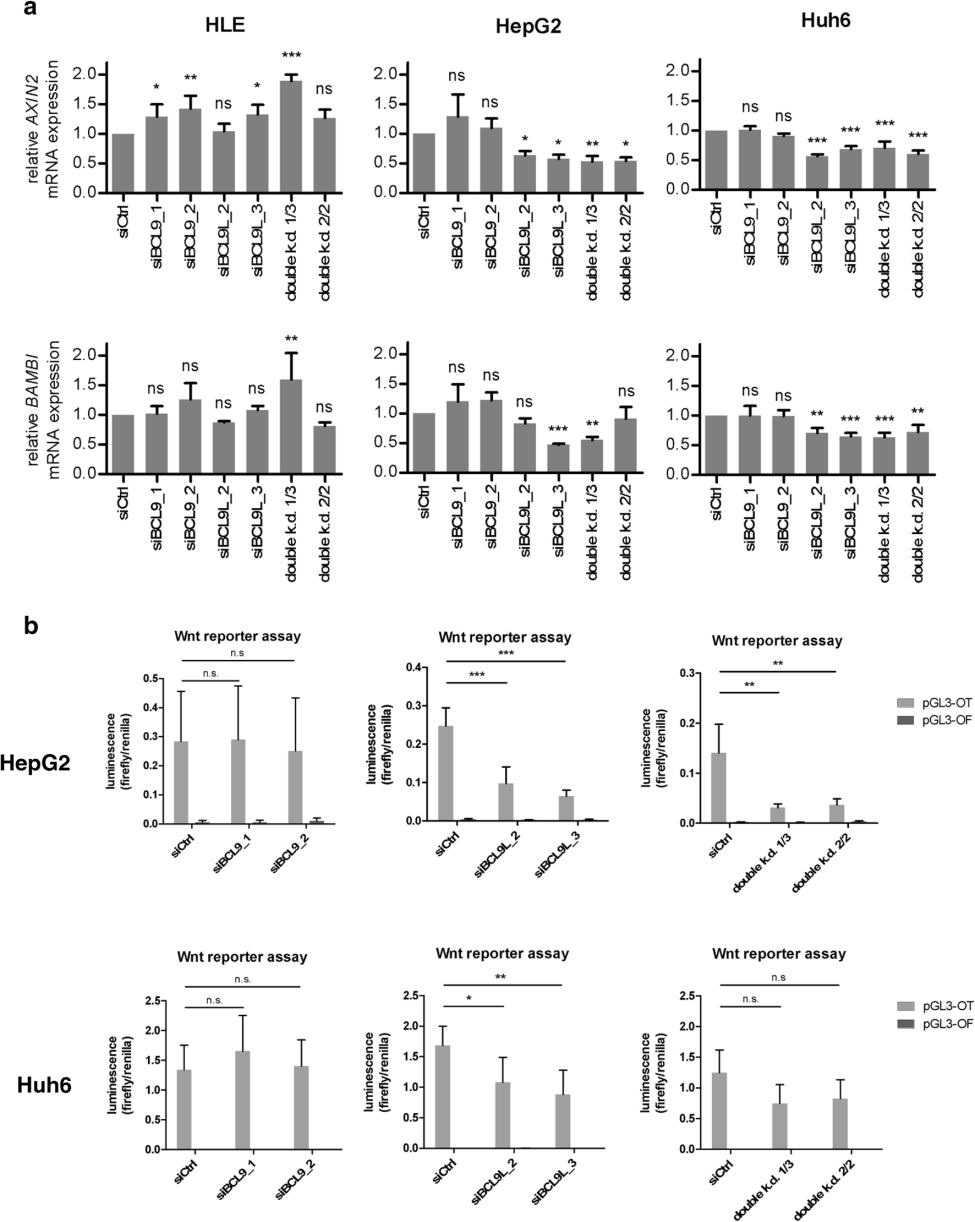
Knockdown of BCL9L or BCL9/BCL9L interrupts Wnt/β-catenin signaling, while knockdown of BCL9 alone is insufficient to decrease Wnt/β-catenin activity. **a** Expression analysis of *AXIN2* and *BAMBI* by qRT-PCR using the ΔΔCT method in HLE, HepG2, and Huh6 cells after transfection with siBCL9 and/or siBCL9L. Data are represented as mean ± SD of three independent experiments. *p < 0.05, **p < 0.01, ***p < 0.001, n.s. not significant; one-way ANOVA with Dunnett’s multiple comparison test. **b** Effects of BCL9 and/or BCL9L knockdown on Wnt/β-catenin signaling activity of HepG2 and Huh6 cells was determined by Wnt reporter assays. Cells were co-transfected with combinations of siBCL9 and/or siBCL9L and pGL3-OT or pGL3-OF. For normalization, the renilla luciferase vector pGL4.70 was used in all conditions. Data are represented as mean ± SD of at least three independent experiments. *p < 0.05, **p < 0.01, ***p < 0.001, ns not significant; one-way ANOVA with Tukey’s multiple comparison test

Then, we analyzed the effects of BCL9 and/or BCL9L knockdown on Wnt/β-catenin signaling. In Wnt-inactive HLE cells, knockdown of BCL9 and/or BCL9L had no effect or even increased levels of Wnt target genes *AXIN2* and *BAMBI* (Fig. 3a). In Wnt-active HepG2 and Huh6 cells, BCL9 knockdown did not affect *AXIN2* and *BAMBI* expression. In contrast, knockdown of BCL9L alone or in combination with BCL9 reduced *AXIN2* and *BAMBI* expression in HepG2 and Huh6 cells. We further aimed to confirm these findings by performing Wnt reporter assays in Wnt-active HepG2 and Huh6 cells. As there is no detectable Wnt signaling in HLE cells (Supp. Figure 4), we did not perform Wnt reporter assays after siRNA-mediated knockdown in this cell line. Again, Wnt signaling activity in HepG2 and Huh6 cells was not affected by knockdown of BCL9 alone, while knockdown of BCL9L and double knockdown of BCL9/BCL9L significantly decreased Wnt signaling activity (Fig. 3b). Our results indicate an interruption of Wnt/β-catenin signaling upon knockdown of BCL9L and BCL9/BCL9L double knockdown, while knockdown of BCL9 alone is insufficient to decrease Wnt/β-catenin activity.

### Functional effects of *BCL9* and/or *BCL9L* knockdown in HCC cell lines

**Fig. 4:**
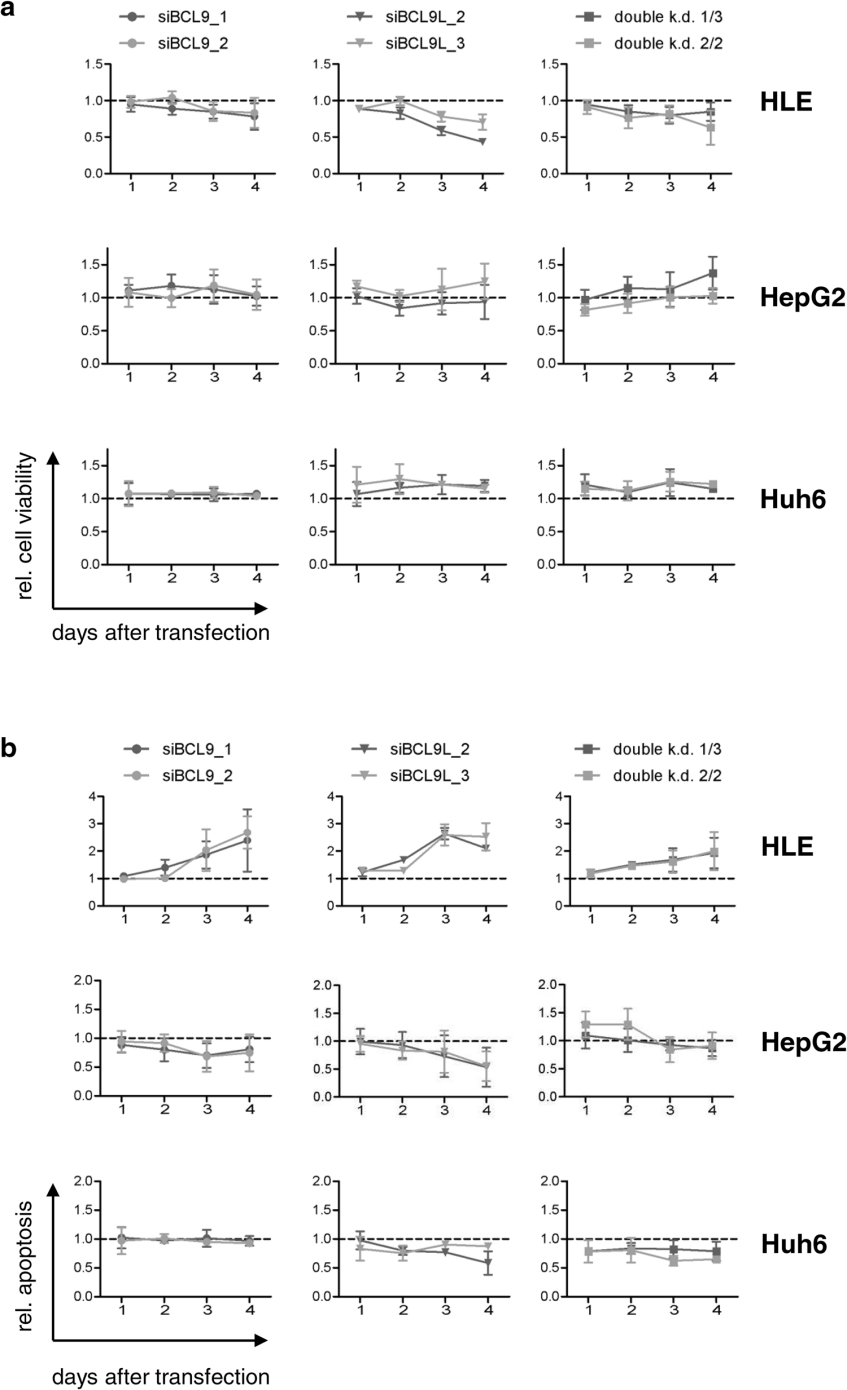
Knockdown of BCL9 and/or BCL9L prevents proliferation and induces apoptosis in HLE cells, but not in HepG2 or Huh6 cells. HLE, HepG2, and Huh6 cells were transfected with 5 nM siRNA against BCL9 and/or BCL9L. Cell viability and apoptosis were determined every 24 h. Data are represented as mean ± SD of at least three independent experiments. **a** Cell viability was analyzed by WST-1 assay and normalized to siControl (dotted line). **b** Apoptosis was analyzed by caspase 3/7 activity and normalized to cell viability and siControl (dotted line)

We next sought to analyze the functional effects of BCL9 and BCL9L by performing cell viability and apoptosis measurements after knockdown of BCL9 and/or BCL9L in HLE, HepG2, and Huh6 cells (Fig. 4). The functional effects of BCL9, BCL9L, and BCL9/BCL9L double knockdown clearly differed between the three tested cell lines. In HLE cells, knockdown of BCL9 and/or BCL9L decreased cell viability and increased apoptosis. In HepG2 and Huh6 cells, however, the BCL9 and/or BCL9L knockdown induced no or only slight effects on cell viability or apoptosis. Thus, functional effects of BCL9 and BCL9L are more pronounced in Wnt-inactive than in Wnt-active HCC cell lines.

### Wnt/β-catenin dependence of *BCL9* and/or *BCL9L* knockdown effects in vitro and in vivo

**Fig. 5:**
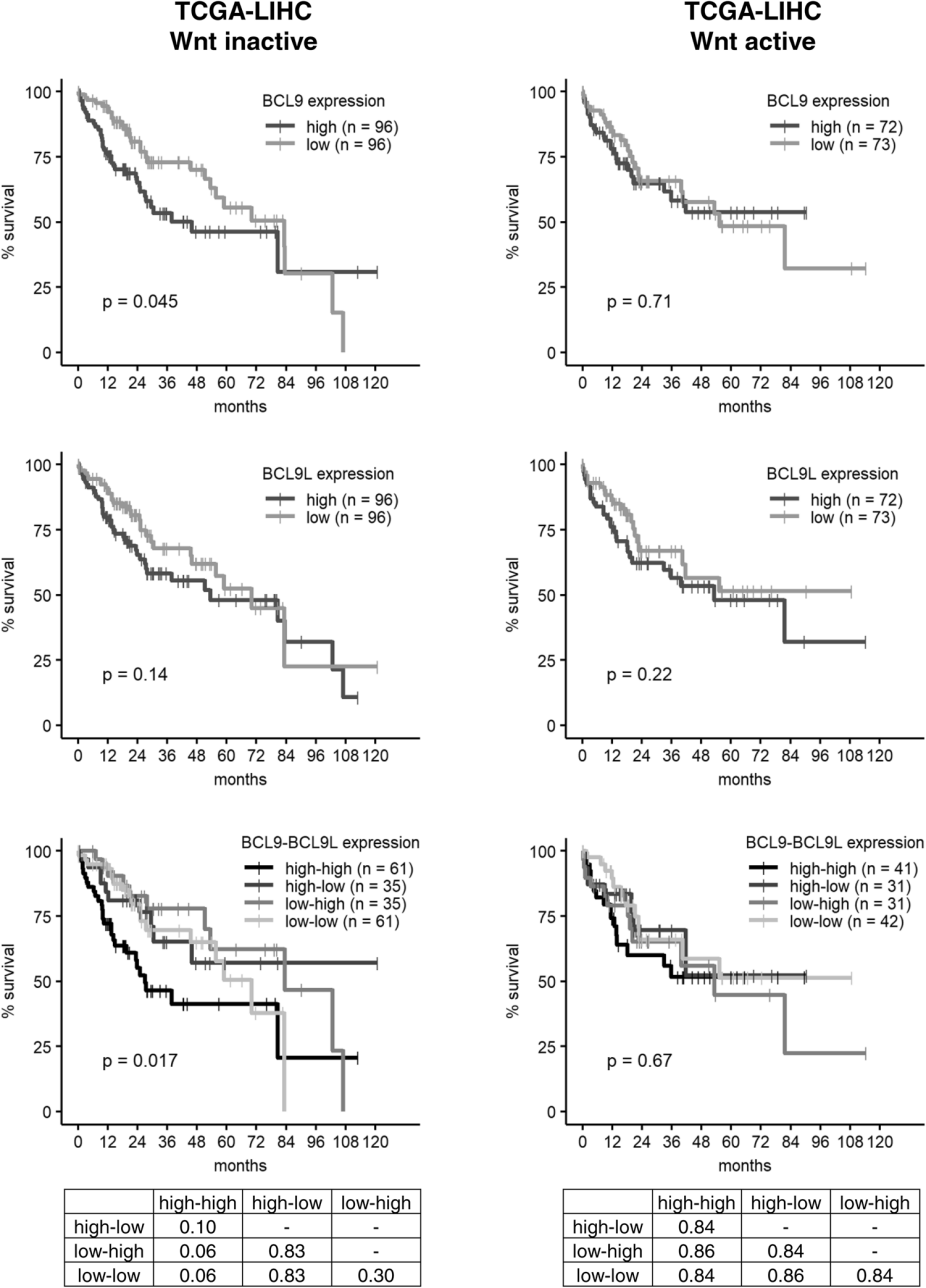
High levels of *BCL9* and *BCL9L* correlate with poor survival only in HCCs with inactive Wnt signaling. *BCL9* and *BCL9L* expression values and survival data of the TCGA-LIHC cohort were retrieved from http://www.oncolnc.org/ [16] and stratified into Wnt-inactive and Wnt-active HCCs according to Sanchez-Vega et al. [17]. *BCL9* and *BCL9L* expression was classified as high (upper median) or low (lower median) and, for combined survival analysis, patients were grouped into the following *BCL9*–*BCL9L* expression groups: high–high, high–low, low–high, and low–low. The tables show the corresponding *p* values for the combined *BCL9*–*BCL9L* survival analysis as determined by log-rank test with Benjamini–Hochberg correction for multiple testing. Survival was analyzed according to Kaplan–Meier

Our in vitro results suggested that functional effects of BCL9 and BCL9L are dependent on the Wnt status of HCC cell lines. This led us to reanalyze the in vivo data sets in the context of Wnt activity. We stratified the TCGA-LIHC cohort into Wnt-inactive and Wnt-active HCCs according to Sanchez-Vega et al. [17] and separately determined how *BCL9* or *BCL9L* expression influences patient survival (Fig. 5). For *BCL9L*, there was no major difference in the survival prognosis of Wnt-inactive and Wnt-active HCCs. For *BCL9*, however, a high expression correlated with poor overall survival only in Wnt-inactive HCCs, but not in Wnt-active HCCs. High expression of both *BCL9* and *BCL9L* also only had a significant influence on patient survival in Wnt-inactive, but not in Wnt-active HCCs.

## Discussion

Alterations of Wnt/β-catenin signaling have been reported in 43–54% of HCCs [4, 17] and are associated with an aggressive cancer phenotype [5]. We show, for the first time, an overexpression of β-catenin transcription complex cofactor *BCL9L* in primary HCC and its association with poor overall survival. We confirmed the previously described overexpression of *BCL9* that is associated with an unfavorable prognosis in human HCCs [8]. Moreover, we observed an increase in *BCL9* and *BCL9L* expression with progressing dedifferentiation and found that *BCL9L* expression was higher in large tumors compared to tumors of smaller size. In fact, the stratification into groups of high and low *BCL9*/*9L* expression for survival analysis led to a slight overrepresentation of high-grade HCCs in the high *BCL9/9L* expression groups (Fig. 2c). This may, to some extent, explain why patients with high *BCL9/9L* expression display an unfavorable prognosis. In addition, to cope with the complexity of HCC, it has to be treated by different therapeutic approaches (e.g., surgery, chemotherapy, embolization, and targeted therapies) or combinations of these, which also may have influenced *BCL9*/*9L* expression and survival analyses. Nonetheless, our results hint towards a potential role of BCL9 and BCL9L as prognostic factors, which has already been proposed for BCL9 in HCC by Hyeon et al. [8].

Whether BCL9 and BCL9L act redundant in humans by compensating each other in the β-catenin transcription complex is an unanswered question. In HCC, only a single study has addressed the effect of a combined deletion of both BCL9 proteins in mouse liver tumorigenesis [11]. This study, however, has not provided answers regarding the redundancy of BCL9 and BCL9L. As both BCL9 proteins share highly conserved homology domains [19], they have been proposed to represent evolutionary duplicates that function in a largely redundant manner at least regarding their interaction with β-catenin [20]. Here, we found that BCL9 knockdown does not affect β-catenin/Wnt signaling, while BCL9L knockdown does. This suggests that BCL9 and BCL9L do not act redundantly in HCC cells. One possible explanation is a one-way compensation of BCL9L for missing BCL9, but not the other way round. Another explanation may be an overall low expression of BCL9, while BCL9L represents the predominant protein in the analyzed HCC cell lines. In this scenario, knockdown of BCL9 would have a minor impact on Wnt signaling activity and low levels of BCL9 would not compensate for BCL9L knockdown. At this time, we can neither confirm nor rule out this hypothesis because comparing protein amounts of Western blots from untreated HCC cell lines might be misleading due to different antibody performance. Our results suggest two different ideas regarding the redundancy of BCL9 and BCL9L, requiring additional information to fully elucidate their roles in HCC.

The majority of Wnt target genes appear to be cell-type specific [21]. Based on results in colon carcinoma cells, *BCL9* and *BCL9L* have been proposed to be Wnt-responsive by de la Roche et al. [12]. In liver cancer cells, however, we did not find any evidence for Wnt responsiveness of *BCL9* and *BCL9L* (Supp. Figure 2). The expression of *BCL9/9L* was neither higher in Wnt-active than in Wnt-inactive HCC cell lines or primary HCCs nor enhanced by activation of the Wnt signaling pathway in HCC cells. Our results do not contradict the results of de la Roche et al., but instead may represent another example of cell-type specific regulation of Wnt target genes.

The hitherto existing results on BCL9’s influence on liver cancer cells are not in line with our observations. Other studies have described growth inhibition upon BCL9 knockdown in Wnt-active Huh6 cells [22] and HepG2 cells [9], as well as an increased Wnt/β-catenin activity upon BCL9 overexpression [9, 23]. Interestingly, we observed that knockdown of BCL9 did not reduce Wnt signaling in Wnt reporter assays and had no effect on the expression of the well-known Wnt target genes *AXIN2* and *BAMBI* in Wnt-active HepG2 and Huh6 cells. In Wnt-inactive HLE cells, however, BCL9 knockdown increased expression of *AXIN2*, hinting towards a Wnt-independent function of BCL9. Moreover, BCL9 knockdown did not influence cell viability or apoptosis of Wnt-active HCC cells, but decreased cell viability and induced apoptosis of Wnt-inactive HCC cells. These in vitro results are in good agreement with our analysis of the TCGA liver cancer data set, showing that high *BCL9* expression correlates with poor survival only in Wnt-inactive human HCCs but not in Wnt-active HCCs. Further experiments may clarify the role of BCL9 in regard to β-catenin/Wnt signaling and its oncogenic effects.

So far, knowledge of BCL9/BCL9L’s molecular function is limited to their role as coactivators in the β-catenin transcription complex. Our results, however, cannot be explained by this mechanism. The reduction of cell viability and induction of apoptosis after BCL9 and/or BCL9L knockdown appear to be independent of the Wnt signaling pathway. This suggests that BCL9 as well as BCL9L exhibit other, yet unknown, molecular functions. Up till now, only a few publications have also proposed a β-catenin-independent role for BCL9 and BCL9L in developmental processes in mice [24, 25]. Pull-down experiments of BCL9 in mouse ameloblasts have indicated binding of BCL9 to proteins involved in exocytosis, membrane-bound vesicles, and extracellular vesicular trafficking [25]. In addition, it has previously been shown that BCL9L activates expression of β-catenin-independent target genes that are required for early intestinal tumorigenesis [7] also hinting towards a more complex role of BCL9 proteins. In line with our results, this provides interesting starting points for further research to elucidate the β-catenin-independent functions of BCL9 proteins.

As deregulated Wnt signaling is a common hallmark of HCC, direct targeting of component proteins of the β-catenin transcription complex appears as an attractive therapeutic approach. BCL9 and BCL9L represent potential molecular targets, because they seem to be dispensable in normal liver homeostasis, but are necessary for liver transformation that is driven by mutant β-catenin [11]. In fact, small molecule inhibitors that target the β-catenin/BCL9 protein–protein interaction interface have already been designed [26]. Our BCL9 and/or BCL9L knockdown experiments underline the possible advantageous effect of downregulation of BCL9 proteins on tumor growth control. Furthermore, our results suggest that targeted therapies aiming at the inhibition of BCL9 proteins may be more beneficial in Wnt-inactive than in Wnt-active HCCs.

## Electronic supplementary material

Below is the link to the electronic supplementary material.

Supplementary material 1 (DOCX 611 kb)
