# Supplementary material for: Wnt status-dependent oncogenic role of BCL9 and BCL9L in hepatocellular carcinoma
Source: Hepatol Int. 2019 Aug 22;14(3):373–84. doi: 10.1007/s12072-019-09977-w (PMC7220899; doi:10.1007/s12072-019-09977-w)
Supplement: Supplementary file 1 — Supplementary material 1 (DOCX 611 kb) [file 12072_2019_9977_MOESM1_ESM.docx]

**Wnt status dependent oncogenic role of BCL9 and BCL9L in hepatocellular carcinoma**

Nicole Huge^1^†, MSc, huge.nicole@mh-hannover.de

Maria Sandbothe^1^†, PhD, maria.sandbothe@posteo.de

Anna K Schröder^1^, MSc, schroeder.anna@mh-hannover.de

Amelie Stalke^1^, PhD, stalke.amelie@mh-hannover.de

Marlies Eilers^1^, eilers.marlies@mh-hannover.de

Vera Schäffer^1^, schaeffer.vera@mh-hannover.de

Brigitte Schlegelberger^1^, MD, schlegelberger.brigitte@mh-hannover.de

Thomas Illig^1^, PhD, illig.thomas@mh-hannover.de

Beate Vajen^1^ ‡, PhD, vajen.beate@mh-hannover.de

Britta Skawran^1^ ‡,PhD, skawran.britta@mh-hannover.de

^1^ Department of Human Genetics, Hannover Medical School, Hannover, Germany

† The first two authors contributed equally to this work

‡ The last two authors supervised this work equally

**Corresponding author**

Dr. Britta Skawran, Department of Human Genetics, Hannover Medical School, Carl-Neuberg-Straße 1, 30625 Hannover, Germany. Tel: +49 511 5324544, fax: +49 511 5324521, email: skawran.britta@mh-hannover.de.

**
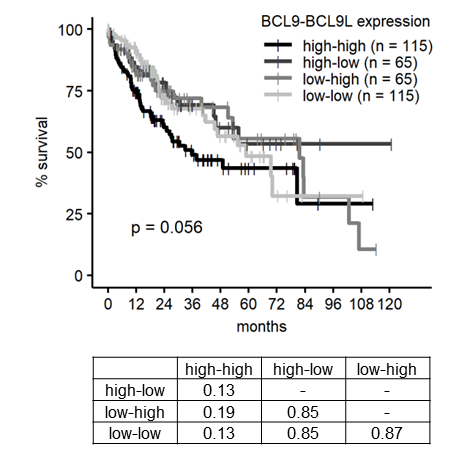
**

**Supp. Fig. 1** High levels of both *BCL9* and *BCL9L* correlate with poor overall survival of HCC patients

*BCL9* and *BCL9L* expression values and survival data of the TCGA-LIHC cohort were retrieved from http://www.oncolnc.org/ [16]. Patients were grouped into the following *BCL9-BCL9L* expression groups: high-high, high-low, low-high, and low-low. The table shows the corresponding *p* values as determined by log-rank test with Benjamini-Hochberg correction for multiple testing. Survival was analyzed according to Kaplan-Meier.

**
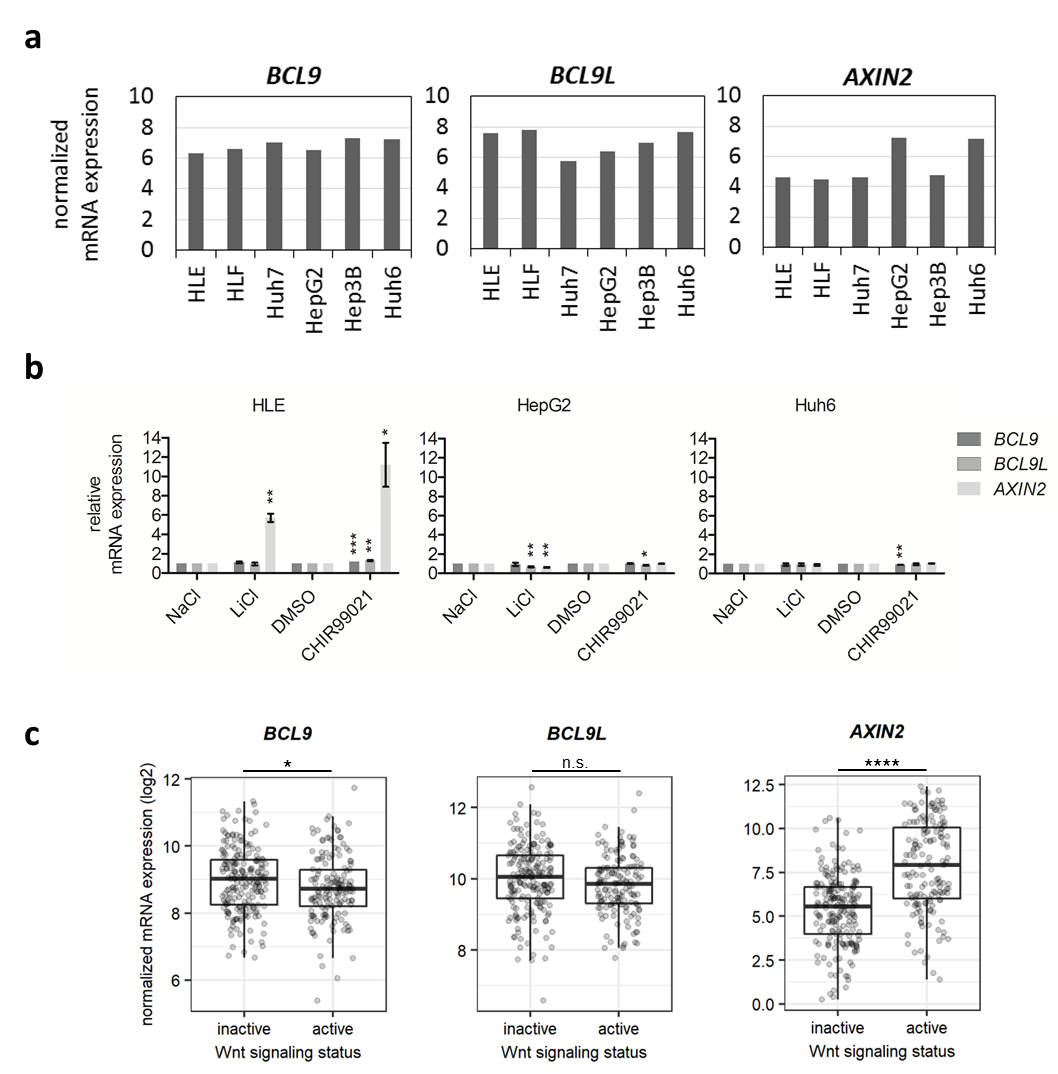
**

**Supp. Fig. 2** Expression of *BCL9* and *BCL9L* in human HCC cell lines and primary HCC is independent of Wnt signaling activation

**a** Microarray-derived RMA-normalized expression levels of *BCL9*, *BCL9L* and *AXIN2*. Data were retrieved from the Cancer Cell Line Encyclopedia (https://portals.broadinstitute.org/ccle) [18].

**b** For activation of the Wnt signaling pathway, HLE, HepG2, and Huh6 cells were treated with 20 mM LiCl (or NaCl, as control) or with 1 µM CHIR99021 (or DMSO, as control) for 24 h. Expression of *BCL9*, *BCL9L*, and *AXIN2* was analyzed by qRT-PCR using the ΔΔCT method. Data are represented as mean ± SD of three independent experiments. **p* < 0.05, ***p* < 0.01, ****p* < 0.001; two-tailed Student’s *t* test.

**c** Expression analysis of *BCL9*, *BCL9L*, and *AXIN2* in primary HCC data from the TCGA-LIHC cohort. Patients were grouped into inactive and active Wnt signaling status according to Sanchez-Vega et al. [17]. **p* < 0.05, *****p* < 0.0001, n.s. not significant; Tukey box-and-whisker plot of a total of 348 samples; 2-tailed Student’s *t* test.

**
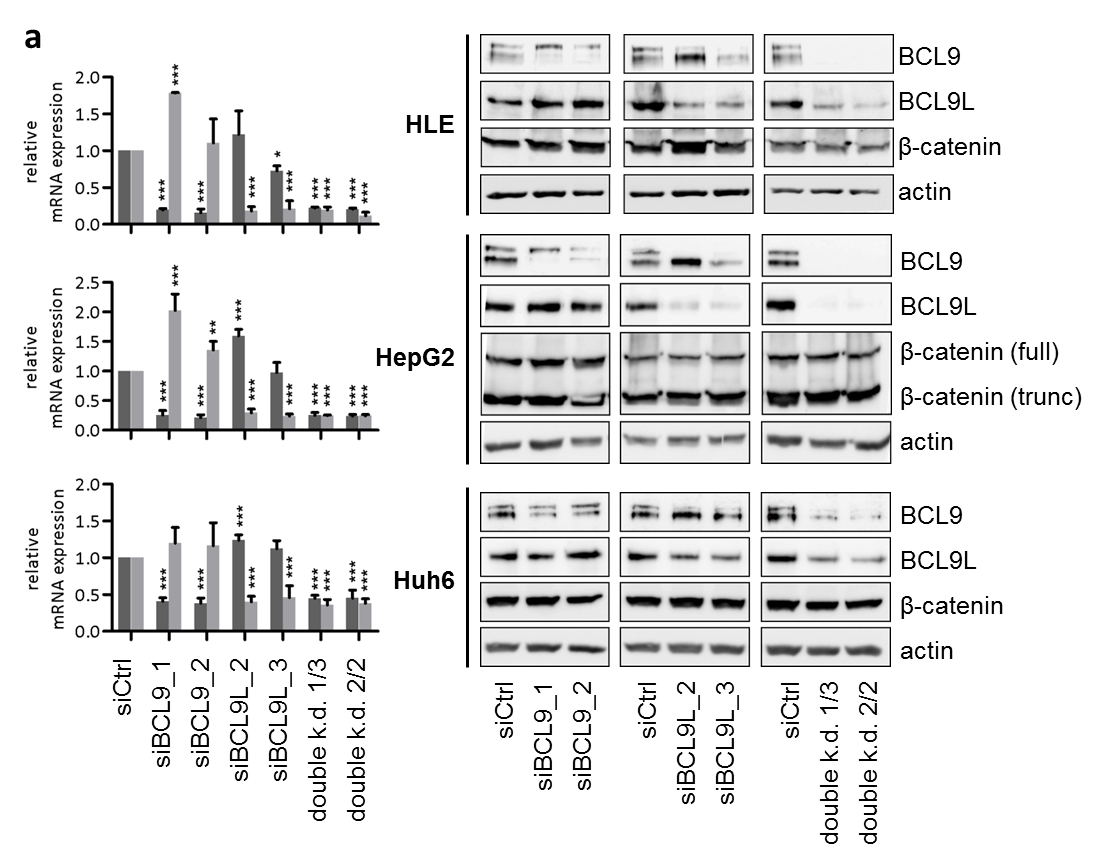
**

**Supp. Fig. 3** Knockdown of BCL9, BCL9L or BCL9/BCL9L in HLE, HepG2, or Huh6 cells

For siRNA-mediated knockdown of BCL9 and BCL9L or BCL9/BCL9L double knockdown, HLE, HepG2, and Huh6 cells were transfected with 5 nM siRNA against BCL9 and/or BCL9L for 48 h.

**a** *BCL9* and *BCL9L* expression was analyzed by qRT-PCR. Results were normalized to the non-targeting control siRNA (siCtrl). Data are represented as mean ± SD of three independent experiments. **p* < 0.05, ***p* < 0.01, ****p* < 0.001; 1-way ANOVA with Dunnett’s multiple comparison test.

**b** Protein expression analysis of BCL9, BCL9L, and β‑catenin by Western blot. Actin was used as loading control.


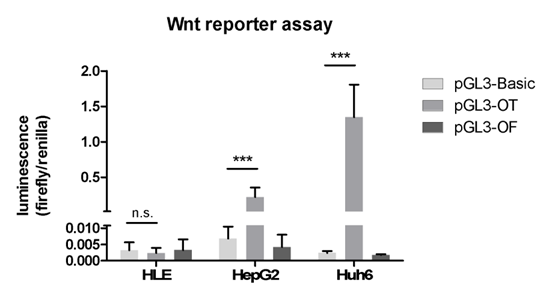


**Supp. Fig. 4** HLE cells show no detectable Wnt signaling activity, in contrast to HepG2 and Huh6 cells

Basal Wnt/β‑catenin signaling activity of HLE, HepG2, and Huh6 cells was determined by Wnt reporter assays. Cells were co-transfected with pGL3-basic, pGL3-OT, or pGL3-OF and renilla luciferase vector pGL4.70. In contrast to HLE, the two Wnt-active HCC cell lines HepG2 and Huh6 show strong β‑catenin/Wnt signaling activity. Data are represented as mean ± SD of at least three independent experiments. ****p* < 0.001, n.s. not significant; 1-way ANOVA with Tukey’s multiple comparison test.
